# Multimodal Emotion Detection via Attention-Based Fusion of Extracted Facial and Speech Features

**DOI:** 10.3390/s23125475

**Published:** 2023-06-09

**Authors:** Dilnoza Mamieva, Akmalbek Bobomirzaevich Abdusalomov, Alpamis Kutlimuratov, Bahodir Muminov, Taeg Keun Whangbo

**Affiliations:** 1 Department of Computer Engineering, Gachon University, Seongnam-si 13120, Republic of Korea; mamiyeva.dilnoza@gmail.com (D.M.);; 2 Department of AI. Software, Gachon University, Seongnam-si 13120, Republic of Korea; 3 Department of Artificial Intelligence, Tashkent State University of Economics, Tashkent 100066, Uzbekistan

**Keywords:** CNN, multimodal emotion recognition, facial feature, speech feature, attention mechanism

## Abstract

Methods for detecting emotions that employ many modalities at the same time have been found to be more accurate and resilient than those that rely on a single sense. This is due to the fact that sentiments may be conveyed in a wide range of modalities, each of which offers a different and complementary window into the thoughts and emotions of the speaker. In this way, a more complete picture of a person’s emotional state may emerge through the fusion and analysis of data from several modalities. The research suggests a new attention-based approach to multimodal emotion recognition. This technique integrates facial and speech features that have been extracted by independent encoders in order to pick the aspects that are the most informative. It increases the system’s accuracy by processing speech and facial features of various sizes and focuses on the most useful bits of input. A more comprehensive representation of facial expressions is extracted by the use of both low- and high-level facial features. These modalities are combined using a fusion network to create a multimodal feature vector which is then fed to a classification layer for emotion recognition. The developed system is evaluated on two datasets, IEMOCAP and CMU-MOSEI, and shows superior performance compared to existing models, achieving a weighted accuracy WA of 74.6% and an F1 score of 66.1% on the IEMOCAP dataset and a WA of 80.7% and F1 score of 73.7% on the CMU-MOSEI dataset.

## 1. Introduction

Emotions are multifaceted psychological phenomena that permeate interpersonal interactions and have far-reaching effects on people’s actions [1]. Recognizing and understanding others’ emotions while communicating is crucial for meaningful conversations. Emotion recognition based on a single modality, such as facial expressions or voice, is difficult and frequently inaccurate [2,3]. Multimodal emotion recognition [4,5] has been developed to address this restriction. The goal of multimodal emotion recognition is to enhance the reliability of emotion identification systems by including data from many modalities, such as facial expressions [6,7], spoken words [8,9,10], and text [11,12]. A person’s emotional state can be captured more accurately by combining many senses. This has sparked a renewed push in recent years to create multimodal systems to identify human emotions.

Facial and vocal expressions are two key components in the process of identifying emotions. Speech transmits information about the tone, prosody, and substance of communication, whereas facial expressions provide visual indications of the emotional state of a person. Using facial expressions alone provides various obstacles and constraints in spite of the fact that they are an important modality for identifying emotions. Other factors, such as lighting, occlusion, and head movement, may also have an impact on face emotions. Furthermore, facial expressions are not always visible since people may try to disguise their emotions, either intentionally or unintentionally. As a result, some phrases may be disregarded. Unfortunately, the accuracy of speech-based emotion identification systems may be impacted by the fact that people have varying accents, dialects, speech speeds, and pronunciations. It is also difficult to capture all important elements for reliable emotion detection since emotions may be expressed via several parts of speech, including prosody, intonation, and lexical content. Therefore, it may be impossible to reliably identify complex emotions, such as mixed emotions, including many states of feeling, based just on facial and vocal expressions. To give complete and accurate insight into a person’s emotional state, it is often important to use both characteristics together.

In the case of multimodal emotion recognition from facial and speech features, deep neural networks [13,14,15] have been employed to extract relevant features from each modality. Networks learn to extract features relevant to emotion classification. For example, in facial expression analysis, deep neural networks can learn to extract features such as facial landmarks [16], head poses, and eye gaze directions. Similarly, deep neural networks may learn to derive characteristics such as pitch, amplitude, and spectral information from speech. However, the complexity of the system in multimodal emotion recognition can be computationally challenging, requiring significant resources and time to train and execute deep neural networks. In addition, overfitting may occur in deep neural networks, which can result in a poor data performance that has never been observed before. The complex and ever-changing characteristics of emotions may also make it difficult for deep neural networks to recognize them reliably under real-world circumstances. By narrowing their focus on the most relevant aspects of the input data, attention mechanisms [17,18,19] have been proven to boost the effectiveness of deep neural networks. This is crucial because not all sensory modalities contribute equally to emotion categorization. Improved emotion detection accuracy may result from the system’s use of the attention mechanism [20,21,22] to zero down on and prioritize the most important modalities. Facial expressions and vocal characteristics may have various feature vector dimensions when multimodal emotion recognition is addressed. Attention mechanisms can be used to equalize the dimensions of feature vectors before feeding them into a neural network, which can lead to more accurate emotion recognition.

In this work, we applied an attention mechanism to improve multimodal emotion identification through vocal and facial characteristics. The suggested emotion recognition system in this research has two primary components: speech feature encoder and facial feature encoder. The facial feature encoder extracts high and low facial features from images using a convolutional neural network (CNN), whereas the speech feature encoder uses the Mel-frequency cepstral coefficients (MFCCs) via CNN to ensure a stable training process for the spectral and time information and waveform features to avoid losing important information when dealing with speech data of varying lengths. After extracting the features from the two modalities, we used an attention mechanism to select the most important features for each modality. The attention mechanism takes the feature vectors from the facial and speech modalities as input and computes an attention weight for each feature vector. The attention weight reflects the importance of each feature vector in the overall emotion recognition task. The attention weights for facial and speech features are combined using a fusion network to create a multimodal feature vector. This multimodal feature vector contains the most important features from both modalities and is fed to a classification layer for final emotion recognition. Our experimental results demonstrate the reliability of the suggested system on the IEMOCAP and CMU-MOSEI datasets. The proposed model has the potential to be used in various applications, including affective computing, human–robot interaction, and mental health diagnosis.

This work contributed to the area of multimodal emotion recognition in numerous ways:

- This study suggests a novel way for identifying multimodal emotions by bringing together facial and verbal clues with an attention mechanism. This method addresses the shortcomings of unimodal systems and enhances the accuracy of emotion recognition by using valuable data from both modalities.
- Time and spectral information were used to address the challenges posed by varying the length of speech data. This allows the model to focus on the most informative parts of the speech data, thereby reducing the loss of important information.
- Facial expression modalities involve generating low- and high-level facial features using a pretrained CNN model. Low-level features capture the local facial details, whereas high-level features capture the global facial expressions. The use of both low- and high-level features enhances the accuracy of emotion recognition systems because it provides a more comprehensive representation of facial expressions.
- This study improves the generalization of the multimodal emotion recognition system by reducing the overfitting problem.
- Finally, the attention mechanism is effectively utilized to focus on the most informative parts of the input data and handle speech and image features of different sizes.

This article’s remaining sections are structured as follows: In Section 2, recent research related to multimodal emotion recognition, including speech and facial expression modalities, and other DL methodologies that integrate attention mechanisms are discussed. Section 3 and Section 4 present and explain, in detail, the workflow of the proposed multimodal emotion recognition system, and the empirical results of the proposed model, including a performance comparison with benchmark models. Section 5 concludes the paper by summarizing the contributions of the proposed model and discussing potential future directions for improving the proposed system. Finally, a list of recent referenced studies is provided.

## 2. Related Works

In recent years, there has been growing interest in multimodal emotion recognition, driven by advances in deep learning and signal processing techniques. Researchers have proposed and tested various methods [23,24,25,26] to achieve high accuracy in multimodal emotion recognition, and this field has seen significant progress in terms of both accuracy and real-world applications. Human emotions are subjective and may be influenced by elements such as cultural background, personality, and situational context, making multimodal emotion detection challenging. As a result, developing a single model that can reliably anticipate emotions for all circumstances and people is challenging. A number of approaches [27,28] for addressing this issue have been suggested, including customizing multimodal emotion detection algorithms to particular circumstances and users. Inspired by PathNet’s success in multi-task learning, the research [27] presents a meta-transfer learning strategy for emotion identification, testing its efficacy in transferring emotional information across visual datasets and considering its potential for voice signals. Moreover, in terms of model generalization, the authors of the paper [5] suggest a framework for facial emotion recognition that involves a pre-trained spatial transformer network on saliency maps and facial images, followed by a bi-LSTM that incorporates an attention mechanism. This study used only one dataset, which may limit the generalizability of the proposed dynamic fusion model to other datasets. 

Dealing with the diversity of human expressions is a significant obstacle in recognizing facial expressions, making it challenging to create a universal model that can precisely identify emotions in all circumstances and individuals. Thus, this article [29] proposes a multi-modal method for extracting emotion features from facial expression images by combining low-level empirical features with high-level self-learning features obtained through CNNs. The former is extracted from the 2D coordinates of facial key-points, whereas the latter is obtained from the CNNs. Although several methods [30,31] have been proposed for facial expression recognition, there is still room for improvement in terms of accuracy and generalization. 

Moreover, recognizing emotions from speech signals has shown great potential with the use of deep learning-based methods, particularly with the implementation of CNNs and RNNs [32,33,34]. The article [35] proposes an approach for improving speech emotion recognition which enhances speech features by selecting specific subsets of the feature set, applying principal component analysis to these subsets, fusing the resulting features horizontally, analyzing the feature set using t-SNE, and then using the features for emotion recognition.

Most emotion recognition methods use only one of these sources. Emotion recognition models that rely on a single source of information may be easier to implement; however, they are more likely to contain errors and inaccuracies owing to the limited scope of the input data. 

Alternatively, multimodal models have the potential to mitigate the deficiencies of relying on a single information source, thereby improving the accuracy and robustness of emotion recognition. Information overload, which may occur when multiple sources of information are combined in multimodal models, can be addressed using an attention mechanism. There has been different research [20,21,22,36,37,38] on the attention mechanisms used in multimodal emotion recognition. The authors of [20] proposed a fully end-to-end model for multimodal emotion recognition that jointly optimizes both phases by taking raw input data, conducting data restructuring, and learning features automatically through end-to-end training, resulting in improved task-specific feature optimization and the elimination of manual feature extraction. However, it introduces higher computational overhead and potential overfitting. To address these concerns, they integrated a sparse cross-modal attention mechanism and sparse convolutional neural network (CNN) to select relevant features, reduce redundancy, and mitigate noise in the input data. In the article [21], video emotion recognition approach emphasizes representation learning and enhances the encoders of audio and visual modalities using global semantic information from text. Additionally, the approach reinforces the audio and visual feature sequences by integrating complementary information from each modality and employing attentive decision fusion to obtain the ultimate emotion prediction.

In addition, this method [39] eliminates the need to detect and follow facial landmarks in emotion recognition systems based on video, which is a common source of errors, thereby improving the resilience of video processing. In addition, the audio component of the system employs Gaussian mixture models (GMMs) specific to utterances derived from a Universal Background Model (UBM) using MAP estimation.

Overall, attention-based multimodal emotion recognition models provide a solution to overcome the challenge of information overload and adapt to varying input uncertainties, leading to improved accuracy and robustness in emotion recognition. As such, these models represent a significant advancement in the field of affective computing, with potential applications in various domains, including healthcare, education, and entertainment.

## 3. The Proposed System

**Figure 1:**
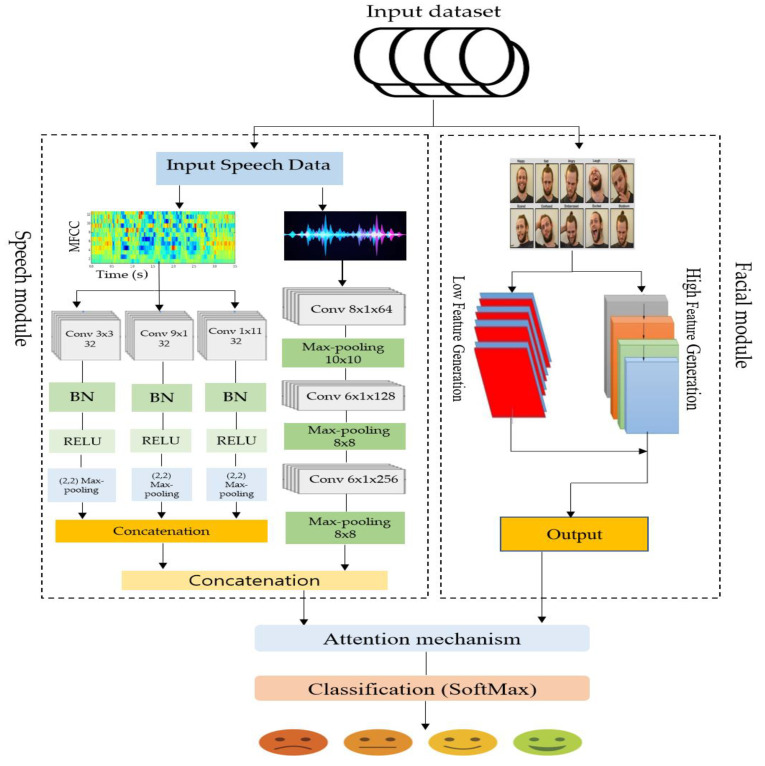
The workflow of the developed emotion recognition system.

Our research presents a new method for emotion detection by combining vocal and facial cues with an attention mechanism. Multiple elements make up the modalities, and they all contribute to the emotion prediction process in their own way. Speech modality is modeled using a convolutional neural network (CNN), whereas image modality is modeled using the ResNet model. An attention mechanism is employed to weigh the importance of specific features in emotion recognition. A detailed illustration of the modeling process is presented in Figure 1, which shows the flow of the various components involved in the model. 

### 3.1. Facial Module

The facial feature extraction module is the first component of the proposed emotion recognition that is responsible for extracting features from facial images. It is designed [40] to encode visual information in the form of static images, such as facial expression, eye gaze, and head pose, into a low-dimensional feature vector that can be further processed by the downstream model for emotion recognition. The facial feature extraction module employs ResNet [41] to capture local and global patterns in facial images and generate high-level abstract features that are discriminative for different emotions. 

**Figure 2:**
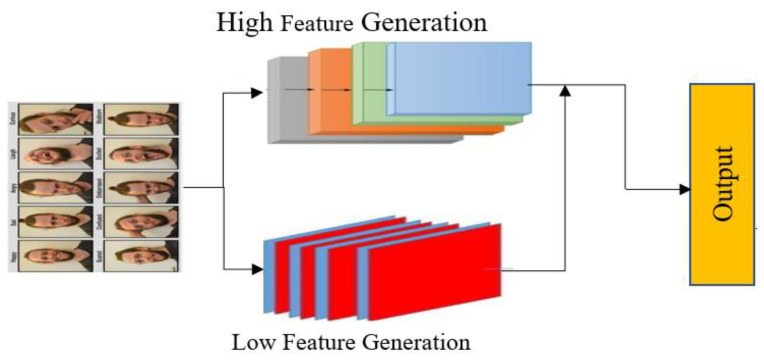
Facial feature extraction model.

The high feature generation pyramid (HFGP) is used for feature extraction and representation from images. It combines shallow and deep features to obtain a more comprehensive representation of the input image. In our facial module (Figure 2), ResNet provides multilevel semantic information for feature maps through its conv4 and conv5 layers. By combining these shallow and deep features, the HFGP can create a more comprehensive representation of the image, capturing both low- and high-level details.

The precision of picture categorization may benefit from this. 

The low feature generation pyramid (LFGP) and convolution layers are stacked alternately as the second phase in the feature creation process. The LFGP is responsible for generating low-level feature maps with a scale different from that of the HFGP, whereas the convolution layers combine the main features and the large output feature map of the preceding pyramid-based layers. This helps further refine and extract relevant features from the input image data. The added feature maps from the low feature generation pyramid are fed into the next convolutional layer, which analyzes and learns the properties of the feature maps from the pyramid layers and considers them as the foundational features of $F_{fo}$.

(1) $$\left[f_{1}^{l},f_{2}^{l},f_{3}^{l},\ldots f_{j}^{l}\right]=\left\{\begin{array}{c} T_{l}\left(F_{fo}\right),\;\;\;\;\;\;l=1\\ T_{l}(P\left(F_{fo,f_{j}^{l-1}}\right),\;l=2,\ldots L \end{array}\right\}$$

Equation (1) indicates that the output multiscale features are obtained by combining the features from the base layer $F_{fo}$ with those from each scale ($f_{j}^{l}$) within each LFGP layer. These combined features are then processed by the HFGP layer ($T_{l}$) and overall HFGP processing (*P*) to produce the final output features. The HFGP method is crucial for creating the final multi-level feature pyramid by fusing features from one level in the network. The 1 × 1 convolution layers utilizing the channels of the input features are used for the compression and coupling operations, which combine the feature maps. The HFGP is particularly effective in detecting small objects because it can rescale deep functions to the same scale as the coupling operation and extract high-decision prototypes for better functional extraction.

#### Low Feature Generation Pyramid (LFGP)

The pyramid network comprises a series of convolution layers with a 2-stride and 3 × 3 kernel. The output of these layers is used as the input for the subsequent convolution layers to generate feature maps. The final layer at each level is selected using the lower convolution layer in the HFGP backbone. To maintain feature smoothness and enhance the learning ability, 1 × 1 convolution layers are added after up-sampling, and a detailed explanation is provided on how the creative sum worked within the top convolution layer network. The multiscale features of the present level are generated by combining the outputs of each convolution layer in both HFGP and LFGP.

### 3.2. Speech Module

The speech module encoder is a key component of the proposed emotion recognition system, which extracts relevant features from the input speech data. To build the speech module, we used a component from our previous study [42] as the basis. The speech module encoder consists of two branches: one for processing the MFCCs and the other for processing the waveform. The MFCC and waveform branches comprise several layers of convolutional units that learn to extract relevant features from the MFCCs and raw waveform signals. The outputs of both branches are then concatenated, and the concatenated vector as a speech module feature is passed with facial module features through a self-attention mechanism that learns to weigh the contributions of each feature module based on its importance in the emotion prediction task. 

#### 3.2.1. MFCC Feature Extractor

CNN blocks of different sizes are designed to facilitate the consolidation of the training stage for spectral and time information. Furthermore, it has been shown empirically [43] that an improvement in the accuracy of predictions is correlated with an increase in the size of the effective area of the CNN. An augmented receptive field size leads to a surge in the number of model parameters, ultimately causing model overfitting, as stated in [44]. Considering the aforementioned objectives, the construction of the model components involves the utilization of three CNNs that are parallelly situated with varying filter sizes. The purpose is to extract different feature maps from the MFCC. The resulting features are then concatenated to form the final output. To compute the MFCC for the input speech data, normalization is first performed, followed by windowing to obtain 64-ms divided frames. Subsequently, Fourier transform is applied to each frame to obtain the frequency components. The next step involves computing an initial set of 40 coefficients for each MFCC frame, using an inverse cosine transform, which is then utilized to train the CNN. The following techniques are used to build the CNN blocks:

- expanding the CNN’s depth by incorporating additional layers
- implementing average pooling or larger stride
- making use of expanded convolutions
- employing a separate convolution on each channel of an input

**Figure 3:**
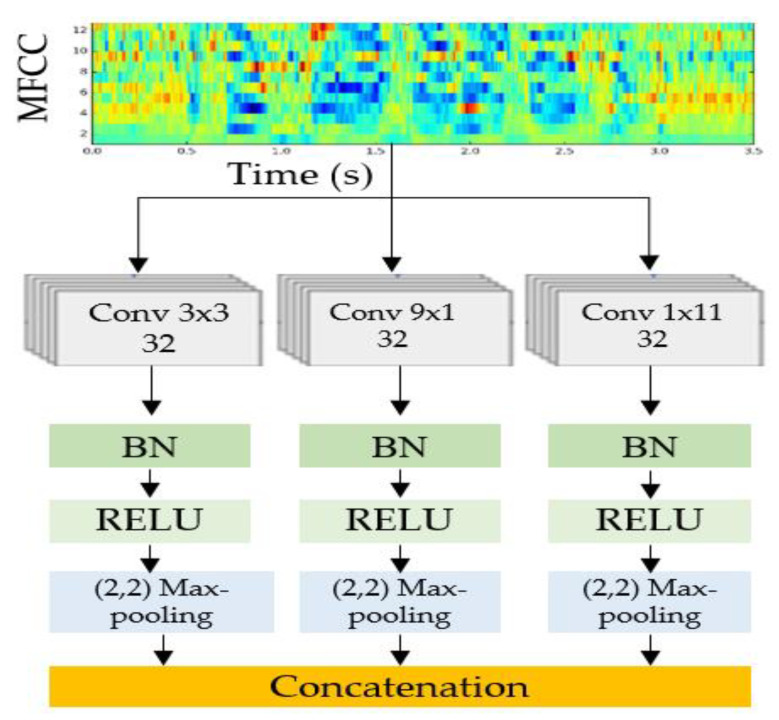
MFCC feature extractor.

By incorporating additional layers and enlarging the size of the convolution kernel (as depicted in Figure 3), a deep CNN is formed to expand the receptive field. Overfitting on complex dimensions is addressed by computing the receptive field individually per dimension, as indicated in reference [43]. The CNN blocks are utilized with different convolution kernel sizes (3 × 3, 9 × 1, and 1 × 11) to capture spectral and time information, resulting in reduced computational complexity and fewer model parameters compared to a single CNN block with a similar effective area size. In the interpretation time, feature-wise actions are performed by batch normalization (BN) where the CNN’s effective area remains constant, and the BN parameters are generated from the effective area and each layer’s activations of the raw speech input. The “spots” of a convolutional kernel are obtained through dilations, resulting in the elimination of kernel weights in the spatial neighboring of samples despite their unchanged number. The convolution process for calculating is performed on the samples by the kernel with a striding factor of “α” when it is diluted. According to reference [43], layers that use dilations make use of an expanded spatial length “α(k − 1) + 1” as the kernel spatial length increases to that value due to dilation. In addition, convolutions can be distinguished based on their channel or spatial dimensions, and these distinguished convolutions have the same receptive field characteristics as their parallel equivalents. To compute the effective area, a kernel size of “3” is used in the 3 × 3 depth-wise convolution, and the resulting encoded MFCC features from each CNN block are merged.

#### 3.2.2. Waveform Feature Extractor

To maintain constancy during the training phase of the proposed model and reinforce its generalization, an attempt is made to incorporate paralinguistic information, as it is believed that the combination of various crucial features, as shown in several developed models [45,46], might lead to better performance. The waveform feature extractor (WFE) comprises a triplet of successive convolutional layers that perform a computation process based on Equation (2), where the input waveform data undergoes the kernel function denoted by f(x).

(2) $$\left(f*x\right)\left(T\right)=\overset{t}{\underset{s=-t}{\sum }}f\left(T\right)\times \left(T-s\right)$$

After obtaining unit variance and zero mean, the input waveform data are partitioned into 20-s intervals and fed to the convolutional layer, which is followed by a max pooling operation that reduces the dimensionality. A key factor in selecting an appropriate pooling size is the size of the convolution kernel. A straightforward empirical approach is adopted, as shown in Equation (3).

(3) $$M=\frac{L-1}{L+X-1}$$

**Figure 4:**
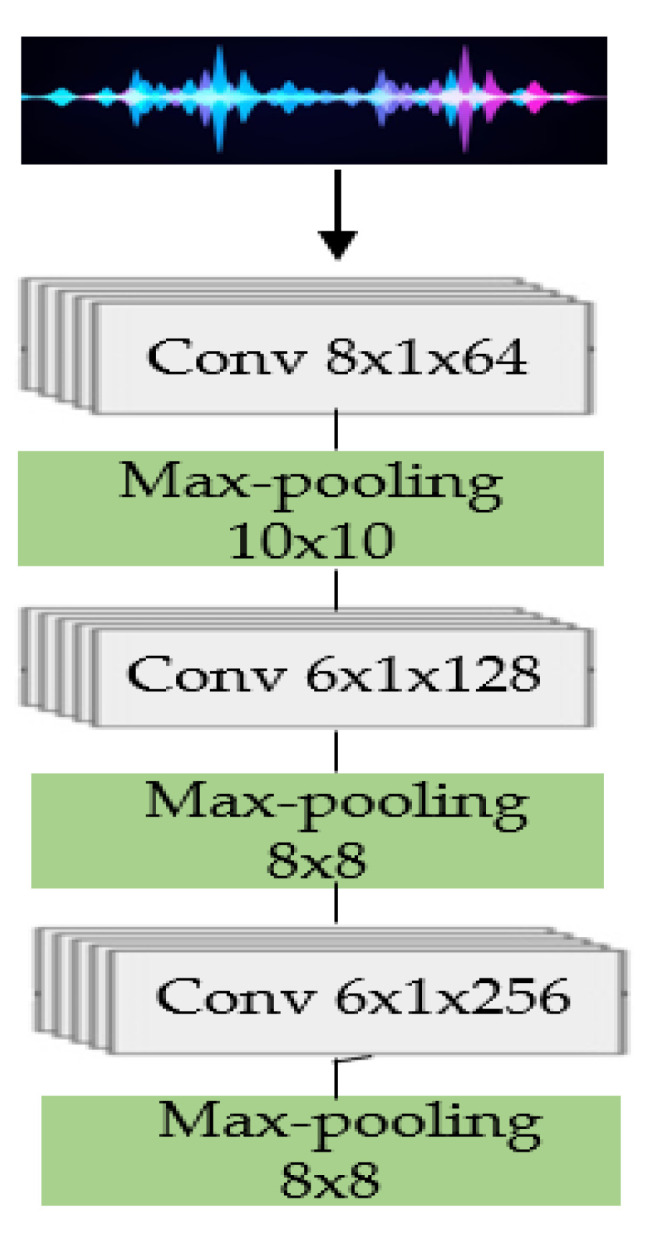
Waveform FE components.

Equation (3) is used to determine an appropriate pooling size based on the convolution kernel size, with “X” denoting pooling size, “L” denoting kernel size, and “M” denoting overlap rate, typically assumed to be approximately 0.5 for hand-crafted features. The complete information is considered by strides, whereas max pooling only concentrates on the most important information and eliminates the irrelevant data; therefore, to prevent acquiring the same characteristics for succeeding frames, the value of “M” should be less than 0.5. As a result, when constructing the WFE architecture, the value of “M” is considered and established as “0.41” for the first convolutional layer and “0.38” for the second and third convolutional layers, as seen in Figure 4.

The WFE structure involves different strides and kernel sizes for the convolution and max pooling. The initial convolutional layer utilizes 64 filters in the temporal domain with an “L” of 8, followed by a down-sampling technique called max pooling, reducing the frame rate with an “X” of 10. The second convolutional layer has a channel size of 128 and an “L” of 6, while the second max pooling layer has a size of 8 and an “M” factor below 0.5. The third convolutional layer has a filter size of 256 and an “L” of 6. A max pooling layer with a dimension of 8 is deployed over the time domain as the final step.

### 3.3. Attention Mechanism

The performance of deep neural networks can be enhanced by an attention mechanism that selectively concentrates on the most important aspects of an object for classification. This enables the attention mechanism to improve the accuracy of the original model. It has been shown that an attention mechanism is useful in natural language processing tasks, such as sentiment analysis [47], where it is used to determine which words and phrases are most important in a sentence to forecast the author’s intent. This has led to its use in other areas, such as multimodal emotion identification, where it has been demonstrated to enhance model performance by focusing attention only on where it will perform the best. The contributions of different modalities may vary in terms of their relevance to sentiment-classification tasks. Some modalities may be more informative and contribute more significantly to a task than others. Therefore, it is crucial to identify and pay more attention to the most relevant modalities while ignoring the irrelevant ones. We have used and partially changed the attention network [48], which takes in both facial and speech modalities and outputs an attention score for each to ensure that only important modalities are emphasized. By assigning more weight to the modalities that contribute the most to the final prediction, this attention mechanism helps the model perform better by zeroing in on the most relevant information. Before being fed into the attention network, the feature vectors of both modalities are scaled to the same size. One approach to achieve this is by using a fully connected layer with a size of “s” to adjust the dimensionality of the feature vectors of all modalities. After normalization to size “s”, the combined facial and speech feature set “*A*” may be written as $A=\left[A_{f},A_{s}\right]$, where $A_{f}$ denotes facial characteristics and $A_{s}$ denotes speech features. Hence, A is a matrix with the dimensions “s by 2”. We calculate the attention weight vector $\omega_{f}$ and the fused multimodal feature vector “$F_{fs}$” as follows:

(4) $$X_{F_{fs}}=\tan h\left(W_{F_{fs}}A\right)$$

(5) $$\omega_{fs}=softmax\left(w_{F_{fs}}^{T}X_{F_{fs}}\right)$$

(6) $$F_{fs}=A\omega_{fs}^{T}$$

where $F_{fs}\in {\mathbb{R}}^{s}$, $\omega_{fs}^{T}\in {\mathbb{R}}^{2}$, $w_{F_{fs}}\in {\mathbb{R}}^{s}$, and $W_{F_{fs}}\in {\mathbb{R}}^{s\times s}$. After computing the fused multimodal feature vector $F_{fs}$ using the attention weight vector $\omega_{fs}$, we used it as an input to the classification layer to perform the final classification of multimodal emotions.

## 4. Experiment

### 4.1. Datasets

#### 4.1.1. The Interactive Emotional Dyadic Motion Capture (IEMOCAP) [49]

**Figure 5:**
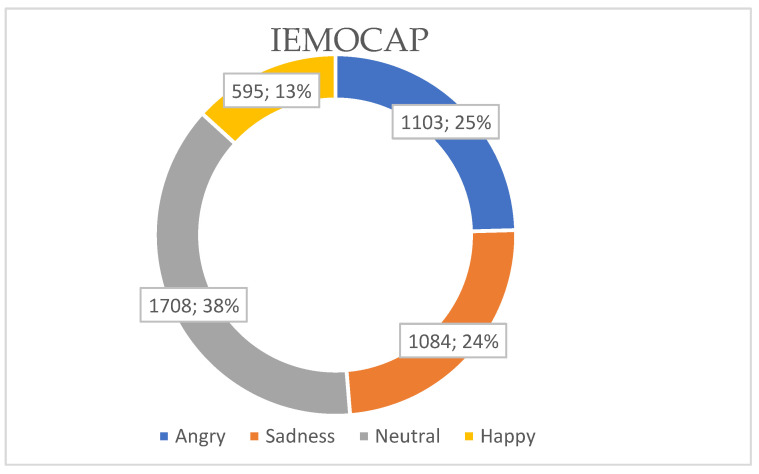
IEMOCAP data distribution.

**Figure 6:**
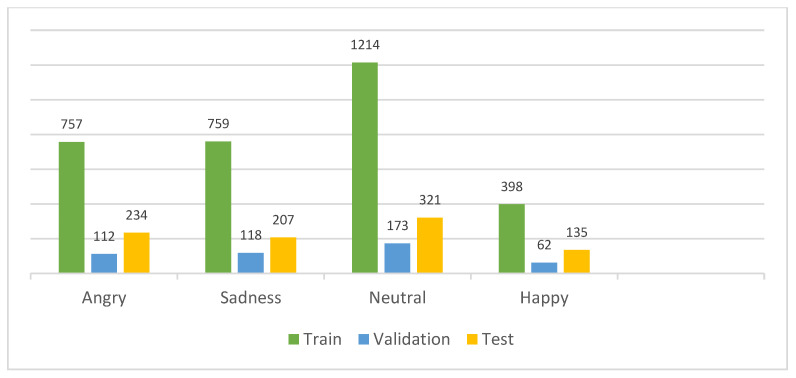
The train/validation and test data samples of the IEMOCAP dataset.

The IEMOCAP dataset is a multimodal database containing audiovisual recordings of spontaneous dyadic interactions between human actors designed for research in multimodal emotion recognition. The dataset includes several modalities, such as audio recordings of speech and motion capture data and video recordings of facial expressions, head movements, and body gestures. The IEMOCAP dataset consists of five sessions conducted by ten distinct speakers, with each session containing recordings from two speakers, comprising one male and one female. The dataset is labeled with four main emotion classes (Figure 5): angry, sad, neutral, and happy. The total dataset consists of 4490 dialogues. There are angry (1103), sadness (1084), neutral (1708), and happy (595) emotional samples. To ensure a fair comparison during the final evaluation of our model using the IEMOCAP dataset, we implemented comprehensive end-to-end training by utilizing the original data reorganized by [20]. To be more specific (Figure 6), we allocated 70% and 10% of the data, which amounts to 3143 and 449 samples, as the training set and validation set, respectively. These samples were extracted from the first four sessions of the dataset, involving eight actors. The remaining 20% of the data, comprising 898 dialogues, was reserved as the test set. These dialogues specifically belong to Session 5 and involve two actors. In contrast to the approach taken in [50], we did not employ 10-fold cross-validation in our study. This decision was based on the impracticality of implementing cross-validation on deep learning models due to the substantial time and computational resources it would demand. 

#### 4.1.2. The CMU-MOSEI [51]

**Figure 7:**
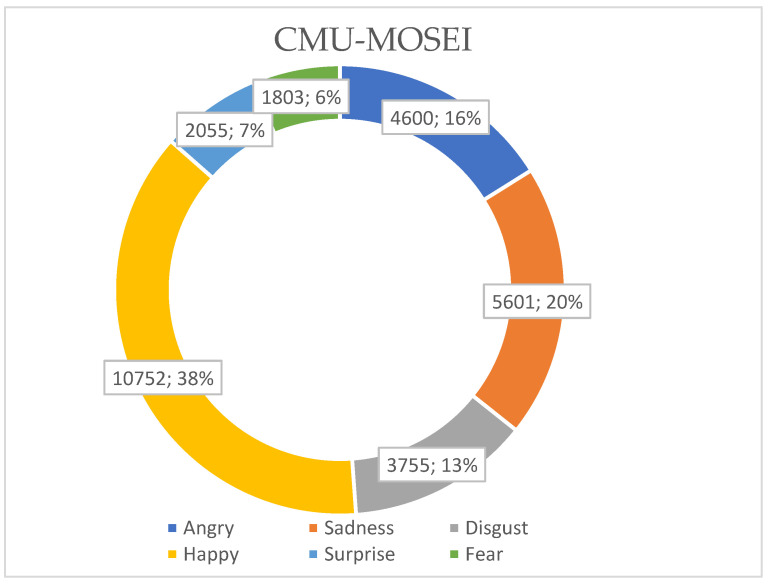
CMU-MOSEI data distribution.

**Figure 8:**
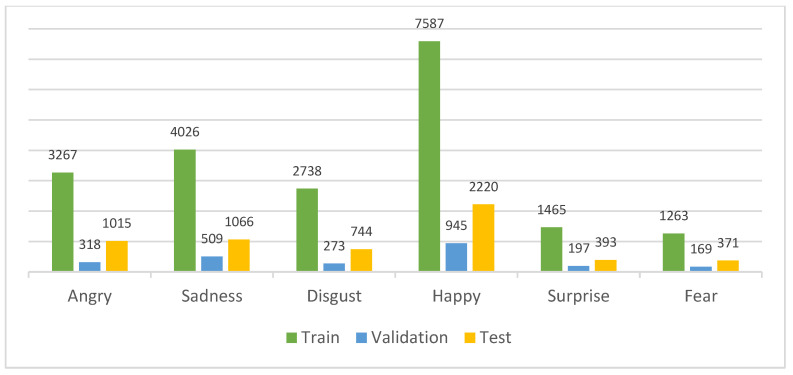
The train/validation and test data samples of the CMU-MOSEI dataset.

The CMU-MOSEI dataset is a large-scale, multimodal dataset for emotion analysis (Figure 7) in the context of conversational video data. The dataset contains more than 23,000 video clips from 1000 sessions with over 1200 participants. The video data is accompanied by speech transcripts, audio features, visual features, and labels indicating the valence and arousal levels. The CMU-MOSEI dataset comprises six emotion classes: anger, happiness, sadness, disgust, fear, and surprise. There are angry (4600), sadness (5601), disgust (3755), surprise (2055), happy (10752), and fear (1803) emotional samples. Similar to the division of the IEMOCAP dataset, we applied the same split to the CMU-MOSEI dataset (Figure 8). For the creation of training and validation sets, we allocated 70% and 10% of the data, amounting to 20,346 and 2411 samples, respectively. The remaining 20% of the data, comprising 5809 samples, was reserved specifically as the test set.

### 4.2. Evaluation Metrics

In our evaluation of the overall model performance, we employed several quantitative metrics to provide a comprehensive assessment. These metrics included the widely used F1 score and weighted accuracy (WA) to account for class imbalances and better capture the average performance across different classes.

WA is a valuable metric that takes into consideration the distribution of emotions within each class. It quantifies the ratio of correctly classified emotions to the total number of emotions belonging to a specific class. By considering the relative importance of each class, WA provides a more accurate representation of performance in scenarios where certain classes may have more instances than others.

By utilizing these metrics in our evaluation, we aimed to gain insights into the model’s ability to accurately classify emotions across different classes, considering both individual class performance and overall class distributions. The formulas for the F1 score and WA metrics are as follows: [provide the formulas for the specific metrics].

(7) $$F1=\frac{2\mathrm{TP}}{2\mathrm{TP}+\mathrm{FP}+\mathrm{FN}}$$

(8) $$\mathrm{WA}=\frac{\mathrm{TP}\times N/P+\mathrm{TN}}{2N}$$

where N is the total number of negative labels and P is the total number of positive labels. TP represents true positives while TN represents true negatives.

### 4.3. Implementation Details

The implementation of the proposed approach involved the utilization of specific software, hardware configurations, and model parameters, as depicted in Table 1.

### 4.4. Experimental Performance and Its Comparison

To provide an indication of the degree to which the proposed system is superior to those offered by the competition, we contrasted it with the criteria listed below. We selected the approaches for comparison based on the datasets. The results of our predictions are shown in Table 2. The selected and proposed systems are viable models for multimodal emotion recognition tasks. However, our system has outperformed the selected models in the MER tasks, particularly when combined with semantic information.

1. Wenliang et al. [20] completely constructed an end-to-end multimodal emotion recognition model that links and optimizes the two stages simultaneously.
2. Xi et al. [21]’s emotion identification from face video includes a semantic improvement module that guides the audio/visual encoder with text information, followed by a multimodal bottleneck transformer that reinforces audio and visual representations via cross-modal dynamic interactions.
3. Multimodal transformer [22]. The suggested technique intends to improve emotion identification accuracy by deploying a cross-modal translator capable of translating across three distinct modalities.

Table 2 refers to the performance evaluation of a system on two different datasets—IEMOCAP and CMU-MOSEI. It is evident that the system surpasses those models in terms of performance. Specifically, on the IEMOCAP dataset, the system achieves a weighted accuracy WA of 74.6% and an F1 score of 66.1%. The WA is a metric that calculates the accuracy of predictions, taking into account the imbalance of the dataset, whereas F1 score is a harmonic mean of precision and recall, which measures the accuracy and completeness of the predictions. Similarly, on the CMU-MOSEI dataset, the system achieves a WA of 80.7% and F1 score of 73.7%, both of which are higher than the existing models evaluated on the same dataset. 

**Figure 9:**
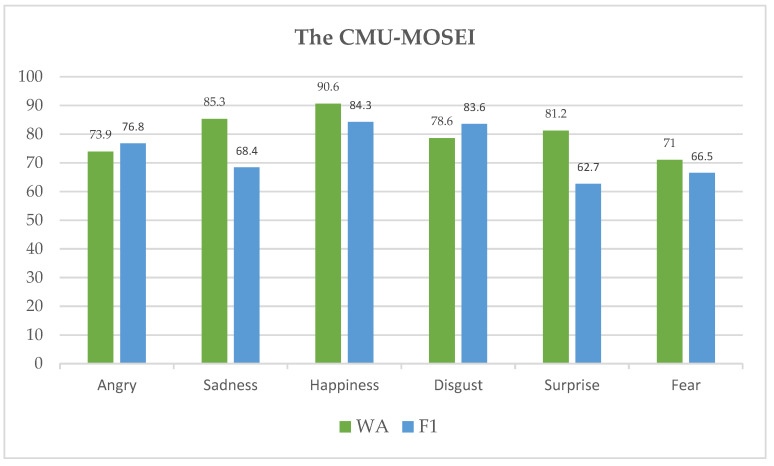
WA and F1 rates for each emotion class of the CMU-MOSEI dataset.

According to Figure 9, the suggested system achieved 90.6% accuracy in WA on the CMU-MOSEI dataset for the happy emotion. It also had excellent accuracies for sadness and surprise, with 85.3% and 81.2%, accordingly. The model performed poorly in identifying angry and fear classes, with 73.9% and 71.0% accuracy, respectively. On the other hand, the proposed approach had the highest F1 rates for the emotions of happiness and disgust, earning 84.3% and 83.3%, respectively. Both surprise and fear were rated lower by the model on the F1 scale, with scores of 76.8% and 66.5%, for instance. 

**Figure 10:**
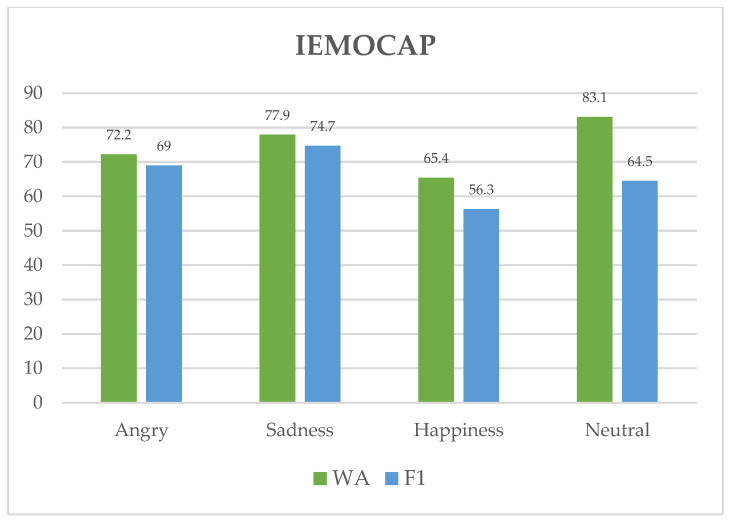
WA and F1 rates for each emotion class of the IEMOCAP dataset.

In the IEMOCAP dataset scenario, we can observe that the system performs inconsistently across distinct feelings. For instance, in Figure 10, when it comes to identifying the emotion “neutral”, WA has a score of 83.1, suggesting that it does an excellent job. The system also shows effective recognition of sadness with a pretty high WA score of 77.9. Moreover, the system displays a relatively high level of accuracy in recognizing sadness (74.7) and angry (69.0) emotions, as shown by its high F1 scores. However, for the emotions of neutral and happiness, the system’s F1 score is lower, with rates of 64.5 and 56.3, respectively. This suggests that the system may have more difficulty recognizing these emotions compared to the others.

**Figure 11:**
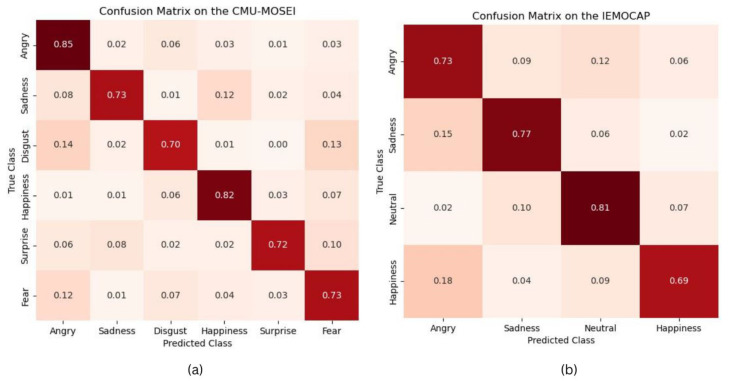
Confusion matrices on the (**a**) CMU-MOSEI and (**b**) IEMOCAP datasets.

We evaluated the performance of the proposed model on the CMU-MOSEI and IEMOCAP datasets. The analysis was conducted using a confusion matrix, as illustrated in Figure 11. The model demonstrated an accuracy exceeding 71% for each emotion class, indicating a reasonably high level of classification accuracy. However, it is important to note that the evaluation dataset exhibited an imbalance in the distribution of samples among the emotion classes. This means that certain classes had a larger number of samples compared to others. Consequently, the model had a tendency to misclassify samples into the classes with a greater representation in the training data.

Specifically, in the CMU-MOSEI dataset, the most common type of error occurred when samples were misclassified into the happy classes. This suggests that the model often labeled samples as happy, even if they belonged to a different emotion category. The prevalence of happy samples in the dataset likely influenced the model’s predictions, resulting in this bias.

Similarly, in the IEMOCAP dataset, the most frequent misclassification involved samples being labeled as neutral. This indicates that the model tended to classify samples as neutral, regardless of their actual emotional label. The higher proportion of neutral samples in the dataset likely influenced the model’s inclination to predict this class more often.

In summary, although the proposed algorithm achieved an overall satisfactory accuracy, the imbalanced distribution of samples across emotion classes led to a bias towards classes with a larger number of samples during the classification process. This highlights the importance of considering data balance and implementing strategies to address bias when training and evaluating emotion recognition models.

### 4.5. Discussion

In this research, we introduced an innovative approach for multimodal emotion recognition by integrating vocal and facial characteristics through an attention mechanism. Our proposed system overcomes the limitations of single-modality systems by combining facial expressions and speech features, leading to improved accuracy in recognizing emotions. By leveraging the valuable information from both modalities, our approach offers a more comprehensive and robust solution for emotion recognition.

The use of MFCCs in combination with a CNN enables our model to extract meaningful representations from the speech data, capturing both frequency-based and time-based attributes. This comprehensive representation facilitates the recognition of subtle variations and nuances in vocal expressions, leading to improved accuracy in identifying emotions. By effectively handling speech data of varying lengths, our system avoids the potential loss of important information that may occur when dealing with diverse utterances. This capability enhances the robustness and reliability of our emotion recognition system, ensuring that it can effectively capture and interpret the rich emotional cues present in speech signals.

Furthermore, our approach incorporates both low- and high-level facial features, extracted through a convolutional neural network (CNN), to achieve a comprehensive representation of facial expressions. The extraction of low-level facial features allows our model to capture intricate local details, such as subtle muscle movements, fine-grained changes in facial contours, and microexpressions. These minute details play a crucial role in conveying specific emotional cues. In addition to low-level features, our system also captures high-level facial features that encompass global facial expressions. These features encompass broader facial characteristics, including overall facial configurations, macroexpressions, and the interplay between different facial regions. By integrating high-level features, our system gains a holistic understanding of the facial expression as a whole, allowing it to capture the overall emotional state being conveyed.

The performance evaluation of the system is conducted on two specific datasets, namely IEMOCAP and CMU-MOSEI. While the results on these datasets show promising performance, it is essential to acknowledge that the system’s effectiveness may vary when applied to other datasets. The generalizability and robustness of the system across a wider range of datasets should be further investigated.

The proposed system heavily relies on the attention mechanism to select the most important features from both facial and speech modalities. While this approach helps in focusing on informative parts, it introduces a potential vulnerability. The system’s performance could be significantly affected if the attention mechanism fails to properly identify and assign appropriate weights to relevant features. Possible issues with attention mechanism performance and its impact on overall system accuracy should be considered. It is essential to consider and address any potential biases that might be present in the training data or the model itself. Biases in emotion recognition systems can arise due to imbalanced datasets, cultural or demographic biases, or biases inherent in the training process.

The superior performance of our system on these datasets highlights its potential in various applications such as affective computing, human–robot interaction, and mental health diagnosis. By leveraging both vocal and facial characteristics and employing an attention mechanism, our proposed methodology offers a promising approach for multimodal emotion recognition, contributing to advancements in the field.

## 5. Conclusions

In summary, emotions play a crucial role in human interactions, and there is a growing interest in multimodal emotion recognition that combines different modalities to provide a more comprehensive understanding of an individual’s emotional state. However, recognizing emotions from a single modality is challenging, and deep neural networks have been used to extract the relevant features. Attention mechanisms have been shown to enhance the performance of deep neural networks by focusing on the informative parts of the input data. 

This study proposes a novel multimodal emotion recognition system that integrates facial and speech features using an attention mechanism. By leveraging complementary information from both modalities, the proposed approach overcomes the limitations of unimodal systems and enhances emotion recognition accuracy. The proposed system for handling speech data of varying lengths utilizes time and spectral information in the speech modality, which enables models to concentrate on the most crucial parts of speech data and minimize the loss of important information. We have also utilized our previously proposed CNN model to acquire low- and high-level facial features. The generalizability of the system has been enhanced by mitigating the issue of overfitting. The effectiveness of the model is demonstrated on the IEMOCAP and CMU-MOSEI datasets; it has promising applications in areas such as affective computing, human–robot interaction, and mental health diagnosis. 

Despite the success of this system, there are several challenges and opportunities for further research. For example, designing efficient and scalable attention mechanisms for large-scale datasets remains a major research direction, as well as integrating attention mechanisms with other techniques, such as reinforcement learning or meta-learning. Moreover, integrating personalized recommendation models [52,53] into multimodal emotion recognition will also be a future direction to significantly improve the emotional well-being and quality of life for individuals. 

We believe that addressing these challenges and opportunities will facilitate more advanced and robust multimodal emotion recognition systems.

## Figures and Tables

**Table 1:** Implementation settings.

Software	Programming tools	Python, Pandas, OpenCV, Librosa, Keras-TensorFlow
	OS	Windows 10
Hardware	CPU	AMD Ryzen Threadripper 1900X 8-Core Processor 3.80 GHz, TSMC, South Korea
	GPU	Titan Xp 32 GB
	RAM	128 GB
Parameters	Epochs	100
	Batch size	32
	Learning rate	0.001, Adam optimizer
	Regularization	L2 regularization, Batch normalization

**Table 2:** Model comparisons on the CMU-MOSEI and IEMOCAP dataset.

Datasets	Models	Metrics	
		WA (%)	F1 (%)
IEMOCAP	Xia et al. [21]	−	64.6
	Multimodal transformer [22]	72.8	59.9
	Wenliang et al. [20]	-	57.4
	Our system	74.6	66.1
CMU-MOSEI	Xi et al. [21]	69.6	50.9
	Multimodal transformer [22]	80.4	67.4
	Wenliang et al. [20]	66.8	46.8
	Our system	80.7	73.7

## Data Availability

Not applicable.
